# First person – Daniel Lenthall

**DOI:** 10.1242/bio.061682

**Published:** 2024-09-02

## Abstract

First Person is a series of interviews with the first authors of a selection of papers published in Biology Open, helping researchers promote themselves alongside their papers. Daniel Lenthall is first author on ‘ Lower-limb coordination changes following a 6-week training intervention that elicited enhancements to maximum velocity sprint performance’, published in BiO. Daniel is a Master's in Research student at Leeds Beckett University, and strength and conditioning coach in the lab of Mr Andreas Wallbaum at the University of Bath, Bath, UK, investigating research that bridges the gap between theory and practice, making a meaningful impact beyond academia.


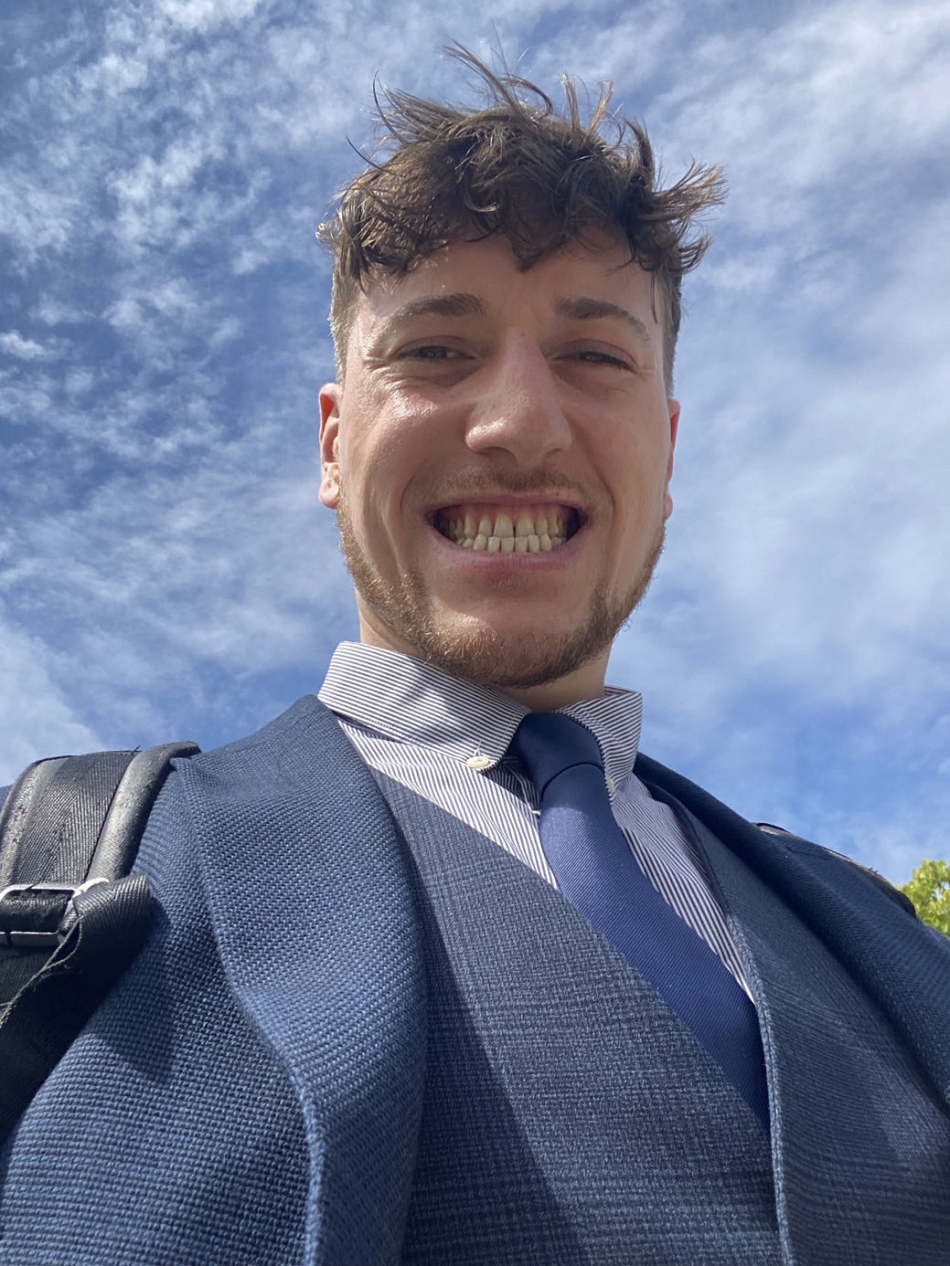


**Daniel Lenthall**

**Describe your scientific journey and your current research focus**

My scientific journey began as an undergraduate at the University of Bath, where I delved into sports biomechanics and its application in strength and conditioning. During this time, I gained hands-on experience through various internships as a biomechanics research assistant. Currently, I am pursuing a Master's in Research at Leeds Beckett University, in collaboration with Hawkins Dynamics and Queen Ethelburga's Collegiate. My research is in evaluating the effectiveness of discrete-point countermovement jump (CMJ) analysis compared to CMJ strategy analysis in detecting periods of heightened neuromuscular fatigue in sport school student-athletes. Additionally, I am investigating how neuromuscular fatigue correlates with athlete well-being among student-athletes. This research aims to enhance our understanding of fatigue management and its impact on athletic performance and well-being.

**Who or what inspired you to become a scientist?**

I didn't come from a scientific background – my parents had little interest in education and did not attend university. Instead, my passion for science sparked when I was around 13 years old, through my involvement in running and weightlifting. I have always had an intense curiosity about how performance can be optimised and thus was naturally drawn to human performance podcasts by Andrew Huberman, Peter Attia, Ben Greenfield, and Andy Galpin. My relentless questioning of “but why?” and “but is there a better way?” revealed that many aspects of performance optimisation are still not fully understood, particularly long-term modifications that characterise improved performance. This realisation drove me to pursue scientific research with the aim of applying findings to enhance sports performance. My goal is to integrate scientific research with practical application, a pursuit that fuels my ongoing scientific inquiry and motivates me to contribute to the field in meaningful ways.

**How would you explain the main finding of your paper?**

In our study, we investigated previously unexplored changes in coordination strategies over a 6-week period of either a multimodal training program or continued participation in sports. We identified key lower-limb coordination modifications that distinguished faster maximal velocity sprinting for those who participated in the multimodal training program. Specifically, we observed that improvements in sprint performance were linked to earlier trail and lead thigh reversals during the stride cycle, as well as more aggressive retraction of the lead limb. Our findings provide empirical evidence that coordination modifications play a crucial role in enhancing sprint performance. The insights gained from this research can be used to refine technical sprinting models and guide the development of more effective training interventions.

**What are the potential implications of this finding for your field of research?**

Our findings on longitudinal coordination modifications characterising enhanced maximal velocity sprint performance have several significant implications for the field. First, they provide a foundation for developing more precise technical models of sprinting, which can be used to better understand and improve sprint mechanics. Additionally, this research can inform the design of targeted training interventions aimed at improving maximal velocity sprint performance. Moreover, the methods from this research can be extended to other populations, such as elite athletes. This broader application could help in exploring how coordination modifications impact performance across different levels of athletic expertise and contribute to more effective training strategies tailored to diverse groups.

**Figure:**
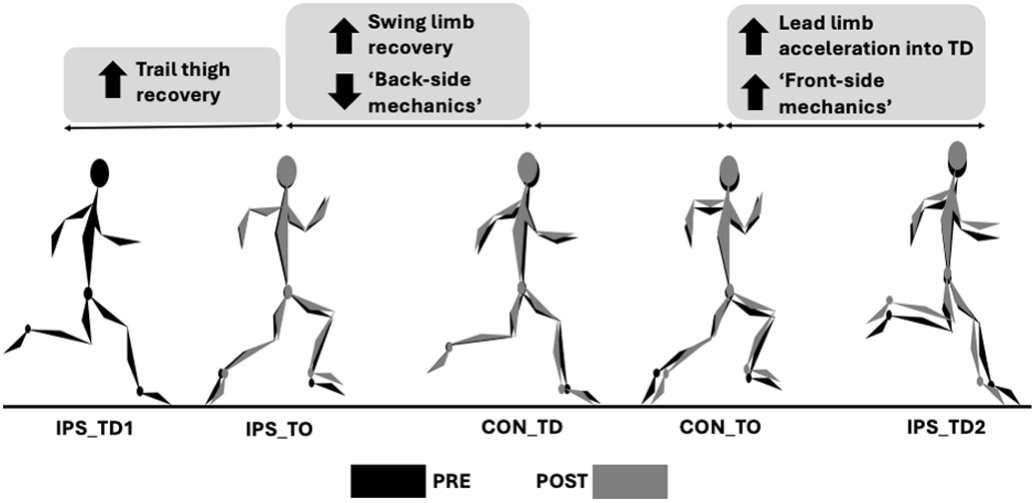
**Visual representation of the primary PRE-POST changes for the intervention group.** TD indicates touchdown; TO, toe-off; IPS, ipsilateral limb; CON, contralateral limb.

**Which part of this research project was the most rewarding?**

The most rewarding part of this research was being part of the first team to investigate longitudinal coordination modifications accompanying participation in a multimodal training intervention, and speculate as to their impact on performance improvement. The novelty of our study inspired to me seek out and implement the most effective analysis methods to provide practical insights for strength and conditioning coaches and practitioners. Additionally, this project offered me my first comprehensive experience across all stages of research – from conducting the literature review and analysing the data, to writing the paper. This hands-on involvement was incredibly fulfilling and provided me with valuable skills and knowledge. Moreover, I developed a strong interest in research that effectively bridges the gap between theory and practice and has the potential to make an immediate impact on sports performance. This experience has been instrumental in shaping the future direction of my research career.

**What do you enjoy most about being an early-career researcher?**

As an early-career researcher, I relish the opportunity to explore largely uncharted areas of scientific inquiry related to optimising sports performance. The freedom and flexibility in this stage of my career allows me to explore new topics and pursue innovative research questions, regardless of the study location or financial cost. I also greatly value the opportunity to learn from seasoned researchers and experts in the field. Engaging with these experienced individuals provides me with invaluable insights and guidance, helping to build a solid foundation for my future career. Their mentorship not only enriches my current research but also shapes my approach to future projects, allowing me to contribute meaningfully to the advancement of scientific knowledge.

…find a passion outside of research – whether it's exercise, a hobby, or something else – that helps you recharge

**What piece of advice would you give to the next generation of researchers?**

I feel I am in the stage of my career where I should still be taking advice rather than giving it. That being said, my primary advice for recent graduates is to seek out mentors who can offer guidance, support, and opportunities for career advancement. I've been fortunate to have two exceptional mentors who have profoundly influenced my career path, and I am deeply grateful to them. Additionally, be sure to bring passion and enthusiasm to every opportunity as making a positive impression can open doors you might not have anticipated. Also, find a passion outside of research – whether it's exercise, a hobby, or something else – that helps you recharge. Lastly, get started! As the old saying goes: “The best time to plant a tree was 20 years ago. The second best time is now”.

**What's next for you?**

My immediate focus is to complete my Master's in Research with a strong emphasis on generating practical insights into fatigue monitoring and athlete well-being among sport school student-athletes. I am excited to share these findings in my forthcoming paper. Additionally, I plan to gain extensive hands-on experience as a strength and conditioning coach at Queen Ethelburga's Collegiate and with elite sport teams in the Leeds area. I am also actively working towards attaining the UK Strength and Conditioning Accreditation by early 2025. Looking ahead, I aim to pursue an applied PhD that integrates research with practical applications in a professional sports setting. I am enthusiastic about the opportunities this path will offer and the potential impact my research can have on the field.
